# RETRACTION: The Impact of Sufentanil versus Remifentanil on Surgical Site Wound Healing in Caesarean Section Primiparas Undergoing Epidural Anaesthesia: A Systematic Meta‐Analysis

**DOI:** 10.1111/iwj.70565

**Published:** 2025-04-18

## Abstract

**RETRACTION:** ChenJ. , LiT. , PanZ. , KeY. , and DingJ. , “The Impact of Sufentanil versus Remifentanil on Surgical Site Wound Healing in Caesarean Section Primiparas Undergoing Epidural Anaesthesia: A Systematic Meta‐Analysis,” International Wound Journal 21, no. 1 (2024): e14377, 10.1111/iwj.14377.37697689 PMC10784625

The above article, published online on 11 September 2023, in Wiley Online Library (http://onlinelibrary.wiley.com/), has been retracted by agreement between the journal Editor in Chief, Professor Keith Harding; and John Wiley & Sons Ltd. Following an investigation by the publisher, all parties have concluded that this article was accepted solely on the basis of a compromised peer review process. The editors have therefore decided to retract the article. The authors did not respond to our notice regarding the retraction.

**RETRACTION:** J. Chen , T. Li , Z. Pan , Y. Ke , and J. Ding , “The Impact of Sufentanil versus Remifentanil on Surgical Site Wound Healing in Caesarean Section Primiparas Undergoing Epidural Anaesthesia: A Systematic Meta‐Analysis,” International Wound Journal 21, no. 1 (2024): e14377, 10.1111/iwj.14377.37697689 PMC10784625

The above article, published online on 11 September 2023, in Wiley Online Library (http://onlinelibrary.wiley.com/), has been retracted by agreement between the journal Editor in Chief, Professor Keith Harding; and John Wiley & Sons Ltd. Following an investigation by the publisher, all parties have concluded that this article was accepted solely on the basis of a compromised peer review process. The editors have therefore decided to retract the article. The authors did not respond to our notice regarding the retraction.

